# No excess risk of colorectal cancer among alcoholics followed for up to 25 years

**DOI:** 10.1038/sj.bjc.6600846

**Published:** 2003-04-01

**Authors:** W Ye, A Romelsjö, K Augustsson, H-O Adami, O Nyrén

**Affiliations:** 1 Department of Medical Epidemiology, Karolinska Institutet, Box 281, S171 77 Stockholm, Sweden; 2 Center for Social Research on Alcohol and Drugs, Stockholm University, Stockholm S106 91, Sweden; 3 Department of Public Health Sciences, Karolinska Institutet, Stockholm S171 76, Sweden; 4 Department of Epidemiology and Harvard Center for Cancer Prevention, Harvard University, Boston, MA 02115, USA

**Keywords:** colorectal cancers, alcoholism, alcohol

## Abstract

We conducted a population-based retrospective cohort study among 179 398 Swedish patients hospitalised for alcoholism from 1970 to 1994, and found no excess risk for colorectal cancers, overall or at any anatomical subsite. Our findings challenge the hypothesis that alcohol intake is a risk factor for cancer of the large bowel.

A role of alcohol in the aetiology of colorectal cancer has long been suspected. However, epidemiological data, as summarised in two reviews (Kune and Vitetta, 1992; Potter, 1999), are conflicting, both with regard to the existence of such an association and to possible variations by anatomical subsites. The disagreement continues in subsequent studies (Gerhardsson de Verdier *et al*, 1993; Goldbohm *et al*, 1994; Glynn *et al*, 1996b; Le Marchand *et al*, 1997; Hsing *et al*, 1998; Munoz *et al*, 1998; Tavani *et al*, 1998; Nagata *et al*, 1999; Sharpe *et al*, 2002). For experimental colon cancer, the tumorigenesis in the right and left colon was affected differently by the level of alcohol consumed (Seitz *et al*, 1998).

If alcohol is indeed a risk factor for colorectal cancer, this association should be easiest to document among subjects with a high and long-term intake, such as alcoholics. However, previous studies of alcoholics have not been large enough to generate stable relative risk estimates by subsite. Moreover, the follow-up time has generally been short or incomplete (Hakulinen *et al*, 1974; Monson and Lyon, 1975; Robinette *et al*, 1979; Schmidt and Popham, 1981; Adami *et al*, 1992; Tonnesen *et al*, 1994; Sigvardsson *et al*, 1996) We therefore conducted a large nationwide retrospective cohort study, based on the Swedish Inpatient Register, to quantify the risk of colorectal cancer among patients hospitalised at least once with a diagnosis of alcoholism.

## PATIENTS AND METHODS

This record-linkage study was based on the Swedish Inpatient Register created by the National Board of Health and Welfare and described in detail elsewhere (Nyren *et al*, 1995). In brief, the register contains administrative and medical data such as hospital department and discharge diagnoses. Ascertainment of cancer incidence was achieved by linkage to the more than 98% complete Swedish Cancer Register, which used the ICD-7 classification throughout the study period. As a separate code for cancer of the anus (ICD7 code 154.1) was first introduced in 1970, our study period began in 1970, rather than 1965 when the Inpatient Register was started.

We identified 193 040 discharge records in the Inpatient Register with a diagnosis of alcoholism (ICD-8=291, 303; ICD-9=291, 303, 305A) during 1970–1994. Record linkage to the nationwide Register of Causes of Death allowed us to identify deaths among the study cohort through 1995. Corresponding linkage to the Emigration Register identified dates of emigration. To remove records with incorrect national registration numbers, which would otherwise contribute person-years at no risk of cancer, we also linked the cohort file to the Register of the Total Population. If a national registration number could not be traced in this register, or in the reports of death and emigration, it was deemed to be of erroneous or incomplete numbers and thus excluded (7425 records). We also excluded 3348 patients with prevalent cancers, since our analysis concerns first primary cancer only. We further excluded 2136 patients who died during the hospitalisation for alcoholism and 733 patients with inconsistencies uncovered during record linkage. Thus, a total of 179 398 patients, 36 568 women and 142 830 men, were entered into the study cohort.

Since the likelihood of being hospitalised for alcoholism may increase if there are also insidious symptoms of a yet undetected cancer, we excluded the first year of follow-up in our study. Thus, follow-up time (person-years) was calculated 1 year after the first recorded (index) hospitalisation for alcoholism until the occurrence of a first cancer diagnosis, emigration, death, or the end of the observation period (31 December 1995), whichever occurred first. To avoid possible ascertainment bias caused by differential autopsy rates, we did not count cancers found incidentally at autopsy. The expected number of cancers was calculated by multiplying the observed number of person-years in age- (in 5-year groups), gender-, and calendar-year-specific strata by the corresponding cancer incidence rates, derived from the entire Swedish population (Ye *et al*, 2001). Relative risk was estimated as the standardised incidence ratio (SIR), defined as the ratio of the observed number of cancers to that expected. The 95% confidence interval (CI) of the SIR was calculated on the assumption that the observed number follows a Poisson distribution (Bailar and Ederer, 1964).

No information was available on severity or duration of alcoholism, nor on treatment given. However, we had information on accompanying codiagnoses at the index or subsequent admissions. Stratified analyses were performed on the presence or absence of liver cirrhosis. Person-years before the first recorded occurrence of an accompanying disease were allocated to the comorbidity-negative strata. We also stratified the analyses by selected cohort characteristics that may influence the results, including gender and follow-up duration.

## RESULTS

The mean age at index hospitalisation among the 179 398 alcoholics (36 568 women, 142 830 men) was 45 years, and the mean duration of follow-up was around 9 years, yielding a total of 1 627 902 person-years at risk (Table 1

**Table 1:** Characterisitics of the alcoholism cohort in the Swedish Inpatient Register 1970–1994 followed up during 1–25 years through 1995

**Characteristics**	**Total**	**Women**	**Men**
No. of patients	179 398	36 568	142 830
Mean age at entry (years)	45.0	43.2	45.5
Years of follow-up, mean	9.0	8.5	9.2
Person-years at risk	1 627 902	312 777	1 315 125
With a codiagnosis of liver cirrhosis (percent)	7236. (4.0)	1714. (4.7)	5522. (3.9)

). Of these patients, 7236 (4.0%) had a codiagnosis of liver cirrhosis at least once during the period up to 1 year before the end of observation.

Based on a total of 929 incident cancers of the colon or rectum *vs* 931 expected, we did not observe any significant overall excess risk of colorectal cancer during 1–25 years of observation (SIR=1.00, 95% CI, 0.93–1.06) (Table 2

**Table 2:** Standardised incidence ratios (SIR) with 95% confidence interval (CI) for colorectal cancers among alcoholics, stratified by selected cohort characteristics^a^

				**Colon**											
	**Colon and rectum**			**Total (ICD7: 153)**			**Right-sided (ICD7: 153.0)**			**Left-sided (ICD7: 153.1–153.3)**			**Rectum (ICD7:154.0)**		
**Cancer site**	**O^b^**	**SIR**	**95% CI**	**Ob**	**SIR**	**95% CI**	**Ob**	**SIR**	**95% CI**	**Ob**	**SIR**	**95% CI**	**Ob**	**SIR**	**95% CI**
Total	929	1.00	0.93–1.06	557	1.00	0.92–1.09	173	0.97	0.83–1.12	265	0.96	0.84–1.08	372	0.99	0.89–1.09
															
*Gender*															
Male	805	1.02	0.95–1.09	470	1.02	0.93–1.11	143	0.96	0.81–1.13	227	0.98	0.85–1.11	335	1.02	0.91–1.13
Female	124	0.89	0.74–1.06	87	0.94	0.75–1.16	31	1.01	0.68–1.43	38	0.86	0.61–1.18	37	0.80	0.56–1.10
															
*Duration of follow-up (years)*															
1–4	260	0.99	0.88–1.12	155	0.99	0.84–1.16	45	0.89	0.65–1.19	80	1.00	0.79–1.24	105	1.00	0.81–1.21
5–9	278	1.00	0.89–1.13	173	1.05	0.90–1.22	58	1.08	0.82–1.40	77	0.92	0.73–1.15	105	0.94	0.77–1.14
10–14	209	0.95	0.83–1.09	129	0.98	0.82–1.17	37	0.87	0.61–1.20	60	0.93	0.71–1.20	80	0.90	0.71–1.12
15–19	156	1.12	0.95–1.31	86	1.04	0.83–1.29	31	1.18	0.80–1.68	39	0.97	0.69–1.33	70	1.24	0.97–1.56
20+	26	0.80	0.52–1.17	14	0.72	0.40–1.21	2	0.33	0.04–1.18	9	0.96	0.44–1.83	12	0.90	0.47–1.58
															
*Liver cirrhosis*^c^															
No	893	1.00	0.93–1.06	536	1.00	0.92–1.09	167	0.97	0.83–1.13	252	0.95	0.83–1.07	357	0.99	0.89–1.09
Yes	36	1.02	0.72–1.42	21	1.00	0.62–1.52	6	0.87	0.32–1.90	13	1.23	0.65–2.10	15	1.06	0.59–1.75

a The first-year observation was excluded.

b Observed number of cases.

c Person-years before the onset of liver cirrhosis were allocated to the liver cirrhosis-negative stratum.

). When examined by separate subsites, a lack of association was evident for colon (SIR=1.00), right-sided colon (SIR=0.97), left-sided colon (SIR=0.96), and for rectal cancer (SIR=0.99). Further stratification by gender, follow-up duration and presence of liver cirrhosis did not reveal any significant excess risk of colorectal cancer, overall or at any anatomical subsite.

## DISCUSSION

This is the largest cohort study to date on risk of colorectal cancer among alcoholics. It enabled detailed analyses by anatomic subsites. This study design offers a number of strengths, including the reduction in potential recall bias. Further, the high quality of Swedish national registers and the consistent use of the unique national registration numbers assigned to each person ensure correct linkage, thereby precluding selective loss to follow-up. One potential concern is the possibility of biased ascertainment or detection of the cancer outcome. A lower diagnostic coverage among alcoholics than in the population at large would entail underestimation of the true relative risk. However, the Swedish health-care system, with low patient fees and equal access to hospital care for everyone, helps allay this concern. Had underascertainment of cancer morbidity been a general problem, we would expect the overall cancer incidence to be low, but this was not the case (data not shown).

Another concern is the lack of information about amount and type of alcohol intake, duration of alcohol abuse before index hospitalisation, and treatment. It precludes a meaningful assessment of dose–response relation, but it is unlikely to affect seriously the overall association with colorectal cancer risk. Imperfect specificity of the alcoholism diagnosis (a high false-positive rate) could conceivably bias a true positive association towards the null. However, the threshold for making an in-hospital diagnosis of alcoholism is generally high. Patients registered with this diagnosis in their case records mostly have a clearly manifested disease. Therefore, the rate of false-positive alcoholism diagnoses in the Swedish Inpatient Register is deemed to be low. A high prevalence of alcoholism in the reference population might be yet another source for underestimation of a true-positive association with colorectal cancer risk. Admittedly, approximately up to 10% of the Swedish adult population is estimated to have problems with alcohol overconsumption (Mutzell *et al*, 1987; Spak and Hallstrom, 1995), but the per capita consumption in Sweden is lower than in most European and American countries (Ramstedt, 2001).

Our most serious concern is the absence of information about cofactors possibly related both to alcoholism and to the cancer outcome. Confounding from such cofactors, for example, smoking (Giovannucci, 2001), low intake of vegetables and fruits (Potter, 1996), folate deficiency (Giovannucci *et al*, 1995b; Glynn *et al*, 1996a; Su and Arab, 2001), and physical activity (Giovannucci *et al*, 1995a), however, would rather tend to inflate the relative risk estimates. We have been unable to identify any factor – except possibly for use of aspirin that is conceivably high among alcoholics and might protect from colon cancer (Thun *et al*, 1991) – that has the potential of introducing negative confounding. We therefore conclude that our negative findings are unlikely to be explained by bias or confounding.

In conclusion, our findings did not support an important role of alcohol in the aetiology of colorectal cancer.
